# The Mediating Role of ICTs in the Relationship Between Pedagogical Innovation and Student Satisfaction in Private Universities in Northern Peru

**DOI:** 10.12688/f1000research.183195.1

**Published:** 2026-06-25

**Authors:** Marilú Trinidad Flores Lezama, Yanet Vanessa Vásquez Romero, Rosario Mercedes Huerta Soto, Pablo Valentino Aguilar Chávez, Ana Elizabeth Paredes Morales

**Affiliations:** 1 Universidad Cesar Vallejo, Trujillo, La Libertad, Peru; 2 Universidad Nacional Santiago Antunez de Mayolo, Huaraz, Ancash, Peru

**Keywords:** pedagogical innovation; technological integration; ICT; student satisfaction; mediation.

## Abstract

**Background:**

Private universities in northern Peru face the challenge of aligning pedagogical innovation with effective technological integration to improve student satisfaction. Most Peruvian studies rely on bivariate analyses and rarely examine the pathways linking pedagogical innovation to satisfaction. This study analyzes whether ICT integration mediates the relationship between pedagogical innovation and student satisfaction at private universities in northern Peru.

**Methods:**

A quantitative, non-experimental, cross-sectional design was applied to 341 undergraduate students from the northern macroregion of Peru (La Libertad, Lambayeque, Piura, and Áncash), selected through stratified probability sampling. Data were collected using a 40-item self-administered online questionnaire rated on a five-point Likert scale, validated by five expert judges (Aiken's V = 0.92), with excellent construct reliability (α = 0.91–0.97; ω = 0.93–0.98; AVE = 0.60–0.71). The mediation model was estimated using Model 4 of Hayes' PROCESS macro with 5,000 bootstrap resamples.

**Results:**

All five hypotheses were confirmed. Pedagogical innovation positively predicted ICT integration (β = 0.856, p < 0.001) and student satisfaction both directly (β = 0.340, p < 0.001) and indirectly through ICT integration (indirect effect β = 0.480; 95% bootstrap CI [0.367; 0.585]), confirming partial mediation with a variance accounted for (VAF) of 58.5%. The model explained 75.6% of the variance in student satisfaction and 73.3% in ICT integration, with large effect sizes and positive predictive validity (Q ^2^ > 0). Importance-performance analysis (IPMA) identified pedagogical innovation as the construct with the greatest potential impact on satisfaction.

**Conclusions:**

ICT integration partially mediates the relationship between pedagogical innovation and student satisfaction, accounting for more than half of the total effect. Pedagogical innovation also exerts a significant direct effect on satisfaction, indicating that non-technological channels — such as human interaction quality, curricular relevance, and meaningful assessment — remain important. These findings suggest that simultaneous and complementary investment in pedagogical innovation and ICT integration, prioritized through IPMA, offers the greatest return for private universities in northern Peru seeking to improve the quality of the student experience.

## 1. Introduction

### 1.1 Contextual framework and problem justification

Today, modern universities find themselves at a crossroads that goes beyond simply incorporating digital devices into the classroom environment; it is a significant challenge because it reaches to the very root of education: the relationships between teachers and students, the formats for producing knowledge, ^1^ ^–^ ^3^ and the standards governing competency assessments in professional training. The COVID-19 pandemic not only accelerated transformations that were already underway but also brought to light deep-rooted inequities within the university system, including the persistence of anachronistic pedagogies based on lecture-style instruction. ^4^ ^,^ ^5^ Now, this convergence of the growing urgency for change and deep structural inequality means that efforts to improve university quality will ultimately fail unless pedagogical innovation is also ensured—such as the application of technology alongside a greater capacity to respond to students’ needs and satisfaction.

It is at this stage that this tension takes on a specific local dimension, with one of its structural characteristics being the gradual admission by private universities in the north of students who are the first in their families to attend college or the children of parents who did not complete their higher education. This potentially vulnerable profile (the quality of the institutional experience) ^7^ ^–^ ^10^ highlights the urgent need for a balanced delivery of educational innovations and relevant technology. Most Peruvian studies are based on bivariate analyses ^11^ ^,^ ^12^ and very rarely delve into the pathways that would lead from pedagogical innovation to student satisfaction, thereby offering insights into the relationship between these two variables.

### 1.2 Pedagogical innovation as a systemic process

Pedagogical innovation is no longer understood as a limited set of techniques, but rather as a systemic process that integrates teaching practices, curricular frameworks, and institutional infrastructures. ^2^ ^,^ ^13^ ^,^ ^14^ From this perspective, active methodologies such as project-based learning, the flipped classroom, and authentic assessment constitute tangible options for a more profound shift in our understanding of learning. ^1^ ^,^ ^15^ ^,^ ^16^ Likewise, the most recent systematic reviews show that the effectiveness of these practices depends on the coherence between the micro, meso, and macro levels of the institution and, of course, on the sustained support provided to teaching. ^17^ ^,^ ^18^

### 1.3 Technological integration: From TPACK to SAMR and the adoption of ICT

Three decades of research on the integration of technology in higher education have yielded coherent and comprehensive theoretical frameworks that converge on a central idea: the presence of devices in no way guarantees pedagogical transformation without intentional instructional design. ^19^ ^,^ ^20^ The TPACK model proposed by Mishra and Koehler ^21^ suggests that effective teaching with technology requires the dynamic articulation of content knowledge, pedagogical knowledge, and technological knowledge. ^22^ ^,^ ^23^ Puentedura’s SAMR model ^24^ ^,^ ^25^ proposes a four-level taxonomy: substitution, augmentation, modification, and redefinition, and empirical evidence confirms that substantial improvements in learning and satisfaction are observed only at the transformational levels. ^26^ ^,^ ^27^ Complementarily, the TAM and UTAUT2 models of technology acceptance ^28^ ^–^ ^30^ provide best practices for understanding students’ adoption of ICT, with an explanatory power of approximately 74% of the variance in the intention-to-use variable. ^31^

### 1.4 Student satisfaction as a multidimensional construct

Student satisfaction has established itself as one of the most widely used indicators for evaluating university quality, not only for its diagnostic value but also for its predictive power regarding persistence, academic performance, and institutional loyalty. ^32^ ^–^ ^34^ It is important to note that its theoretical foundations trace back to Astin’s input-environment-output model, ^7^ ^,^ ^35^ Tinto’s integration theory, ^9^ ^,^ ^10^ ^,^ ^36^ and the conceptualization of student engagement proposed by Kuh and colleagues. ^37^ ^,^ ^38^ A second line of research, originating in services marketing, ^39^ ^,^ ^40^ enriches our understanding by conceiving satisfaction as the confirmation or non-confirmation of preliminary expectations. This research employs a multifactorial model of student engagement that integrates academic quality, institutional resources, social interactions, and the overall educational experience. ^41^ ^,^ ^42^

### 1.5 Current state of research on the mediation of ICT between innovation and satisfaction

The systematic review conducted by Bond et al. ^43^ of 243 empirical studies concluded that innovative pedagogical practices increase student engagement only when accompanied by intentional technological design. García-Peñalvo et al., ^44^ using a sample of 1,326 Spanish students, demonstrated via PLS-SEM that pedagogical quality predicts satisfaction through the perceived utility of ICT. Wang and Chen, ^45^ applying Hayes’ Model 4 to 612 Chinese students, reported a significant partial mediation of technological integration between pedagogical innovation and satisfaction (indirect β = 0.21; 95% CI [0.14; 0.28]). In the Latin American context, Sanabria-Z et al., ^46^ Hernández-Ramos et al., ^47^ and Cabero-Almenara et al. ^48^ confirmed convergent patterns in Mexican, Colombian, and Chilean universities, with mediation percentages ranging from 33% to 41%.

In Peru, Estrada-Araoz et al. ^11^ found a moderate correlation between digital competencies and satisfaction (rho = 0.369); Mamani-Benito et al. ^12^ reported a significant effect of online education on satisfaction (β = 0.52); and Ramírez-Asís et al., ^49^ with 894 students from five universities, demonstrated partial mediation by faculty digital competencies (indirect β = 0.28; 95% CI [0.19; 0.37]). Specifically in northern Peru, Pérez-Cuadros and Romero-Espinoza, ^50^ Vargas-Castañeda and Ortiz-Rojas, ^51^ and Coronel-Wong and Sandoval-Romero ^52^ agree that pedagogical innovation alone does not sufficiently explain student satisfaction, which invites the exploration of formally specified mediating mechanisms.

### 1.6 Objective, hypothesis, and contribution of the study

The overall objective was to analyze the mediating role of technology integration (ICT) in the relationship between pedagogical innovation and student satisfaction at private universities in northern Peru, using Model 4 of Hayes’s PROCESS macro model. ^53^ The hypotheses were: H1, pedagogical innovation positively predicts satisfaction (total effect c); H2, pedagogical innovation positively predicts ICT integration (effect a); H3: ICT integration positively predicts satisfaction when controlling for innovation (effect b); H4: there is a significant indirect effect a × b with a bootstrap CI that excludes zero; H5: the direct effect c’ remains significant, constituting partial mediation. ^53^ ^–^ ^55^ The contribution is threefold: theoretical, by integrating three research traditions that have thus far developed relatively independently (educational change, ICT integration, and university satisfaction); methodological, by applying robust bootstrap inference to a sample from northern Peru that was previously underrepresented; and practical, by offering applicable criteria for internal quality assurance systems. ^19^ ^,^ ^20^ ^,^ ^56^

We also consider the fundamental theoretical framework based on service marketing, incorporating models such as SERVQUAL and SERVPERF into higher education, ^39^ ^,^ ^40^ as this perspective conceives satisfaction as the confirmation or non-confirmation of students’ prior expectations regarding the educational service received, taking into account the dimensions of reliability, responsiveness, assurance, empathy, and tangibles (a model based on five fundamental dimensions). Some recent applications in higher education demonstrate that perceived service quality strongly predicts or explains satisfaction and institutional loyalty, ^32^ ^,^ ^57^ however, the uncritical application of these models to the university has been the subject of significant criticism: comparing the student to the consumer can distort the pedagogical relationship and weaken the transformative dimension of the educational process. ^10^ ^,^ ^41^ This study acknowledges these criticisms and is framed within a multifactorial context that avoids reducing or minimizing satisfaction to a simple or mere immediate gratification.

### 1.7 Conceptual framework of the study

The theoretical synthesis developed in the previous sections translates into the conceptual model underpinning this study, as follows: pedagogical innovation (X) constitutes the independent variable; the integration of ICT (M) acts as a mediator; student satisfaction (Y) represents the dependent variable. The arrows a, b, c, and c’ indicate, respectively, the effect of X on M, the effect of M on Y controlled for X, the total effect of X on Y, and the direct effect of X on Y controlled for the mediator. The theoretical basis of this model rests on four interrelated premises: first, contemporary university pedagogical innovation does not operate independently of technological mediation but is largely realized through it. ^13^ ^,^ ^26^ ^,^ ^58^ Second, high-quality technological integration, as understood in the TPACK and SAMR models, predicts student satisfaction through mechanisms of perceived utility, hedonic motivation, and cognitive engagement. ^21^ ^,^ ^30^ ^,^ ^59^ Third, student satisfaction is a multidimensional construct sensitive to academic quality, infrastructure, and social interactions. ^7^ ^,^ ^9^ ^,^ ^42^ Finally, and fourthly, partial mediation constitutes the most prominent hypothesis from a theoretical standpoint, as pedagogical innovation preserves non-technological channels, as well as the quality of human interaction and the vital importance of the curriculum. ^45^ ^,^ ^55^ The main empirical evidence informing this conceptual model is synthesized in Table 1.

**Table 1.** Summary of the main empirical evidence on the mediating role of ICTs between pedagogical innovation and student satisfaction.

Main conclusion	Design	n	Country	Author (year)
Engagement increases only when the technology is accompanied by intentional instructional design.	Systematic review	243 studies	United Kingdom	Bond et al. ^43^
The quality of teaching predicts satisfaction with the, mediated by the perceived usefulness of ICT.	PLS-SEM	1326	Spain	García-Peñalvo et al. ^44^
Partial mediation; indirect effect β = 0.21 (95% CI [0.14; 0.28]).	PROCESS Model 4	612	China	Wang and Chen ^45^
Indirect effect β = 0.33 through active learning and digital support.	PLS-SEM	1528	Mexico	Sanabria-Z et al. ^46^
Partial mediation; 38% of the total effect is mediated by technological integration.	SEM	489	Colombia	Hernández-Ramos et al. ^47^
Teachers’ and students’ digital competencies jointly predict satisfaction.	Multiple mediation	822	Chile	Cabero-Almenara et al. ^48^
The factor most closely related to satisfaction is perceived pedagogical quality, not ICT resources.	Comparative quantitative	1,113	Spain, Italy, Ecuador	Tejedor et al. ^60^
rho = 0.369 was observed between digital competencies and academic satisfaction.	A correlation was observed	147	Peru (Puno)	Estrada-Araoz et al. ^11^
β effect = 0.52 of online education on satisfaction.	SEM	432	Peru (Lima)	Mamani-Benito et al. ^12^
Partial mediation; indirect effect β = 0.28 (95% CI [0.19; 0.37]).	PROCESS 4 model	894	Peru	Ramírez-Asís et al. ^49^
67% average-high satisfaction; 41% consider that ICTs have a clear pedagogical purpose.	Quantitative	412	Peru (Trujillo, Chiclayo)	Pérez-Cuadros and Romero-Espinoza ^50^

*Note*: Selection of the most representative empirical evidence. The comprehensive review is included in the supplementary material.

## 2. Materials and methods

### 2.1 Design

The study adopted a quantitative approach, basic in nature and explanatory in scope, with a non-experimental, cross-sectional, and explanatory-causal design. ^61^ ^,^ ^62^ The substantive hypothesis was modeled as X → M → Y, with pedagogical innovation as the independent variable, ICT integration as the mediator, and student satisfaction as the dependent variable. The estimation was performed using Model 4 of Hayes’ PROCESS macro ^53^ with a bootstrap of 5,000 resamples. This analytical approach overcomes the limitations of the classic causal procedure by Baron and Kenny, ^63^ which is currently considered statistically conservative and limited when detecting small or moderate indirect effects. ^55^ ^,^ ^64^ ^–^ ^66^

### 2.2 Participants and sampling

The population consisted of undergraduate university students, over 18 years of age, regularly enrolled during the 2026 academic year at private universities in the northern macro-region of Peru—La Libertad, Lambayeque, Piura, and Áncash—with an estimated total of approximately 145,000 students. ^67^ The final sample consisted of n = 341 students, selected using stratified probability sampling by academic program () with proportional allocation based on the population weight of each stratum and simple random selection within each stratum. ^61^ The sample size far exceeds the minimum of 119 observations required to detect medium-sized effects (f ^2^ = 0.15) in a multiple regression with three predictors and a power of 0.80. ^68^ ^,^ ^69^ Students over 18 years of age who were enrolled and had completed at least one academic semester were included; responses with incomplete data or invalid patterns according to Curran's criteria ^70^ were excluded.

### 2.3 Informed consent

Written informed consent was obtained from all participants before their enrollment in the study. The informed consent form explained the purpose of the study, the procedures, the voluntary nature of participation, the confidentiality of the data, and the right to withdraw at any time. It should be noted that no minors participated.

### 2.4 Instrument

A structured, self-administered online questionnaire was administered, consisting of 40 items rated on a five-point Likert scale (1 = strongly disagree /never, 5 = strongly agree/always): 8 items on pedagogical innovation organized into three dimensions (methodological, curricular, and institutional), 16 items on the integration of ICT across four dimensions (infrastructure, effective use, alignment with learning, and digital competencies), and 16 items on student satisfaction across four dimensions (academic quality, resources and services, social interactions, and overall experience). The complete operationalization is presented in Table 2.

**Table 2.** Operationalization of the study variables.

Items	Dimensions	Conceptual definition (reference)	Variable
8	Methodological; curricular; institutional	Intentional process of didactic, curricular, and institutional transformation. ^2^ ^,^ ^14^ ^,^ ^18^	Pedagogical innovation (X)
16	Infrastructure; effective use; alignment; digital competencies	Intentional and pedagogically grounded integration of ICT into teaching and learning. ^21^ ^,^ ^22^ ^,^ ^25^	Integration of ICT (M)
16	Academic quality; resources and services; social interactions; overall experience	Multidimensional subjective assessment of the university experience. ^34^ ^,^ ^41^ ^,^ ^42^	Student satisfaction (Y)

*Note*: All items are rated on a positive 1–5 Likert scale, without reverse coding.

### 2.5 Validity and reliability

Content validity was established through the judgment of five experts—three with doctorates in Education and two in Educational Technology—using a dichotomous rating scale to assess clarity, appropriateness, and relevance. Agreement was assessed using Aiken’s V coefficient with a criterion of V ≥ 0.80 and a 95% confidence interval whose lower limit exceeded 0.70 ^71^ ^,^ ^72^; the mean V was 0.92. Reliability was assessed through a pilot study (n = 30) using Cronbach’s alpha and McDonald’s omega coefficients, with values ≥ 0.70 considered acceptable. ^73^ ^–^ ^76^

### 2.6 Procedure and ethical considerations

The questionnaire was distributed via Google Forms by email and online platforms over a four-week period, with two spaced reminders. Data cleaning removed duplicate records, incomplete responses (more than 10% of items), and invalid patterns. ^70^ The protocol was approved by the Research Ethics Committee of the relevant institution, and informed consent was obtained at the beginning of the questionnaire, in strict compliance with the principles of the Belmont Report and the Declaration of Helsinki. ^77^ Anonymity was ensured by not collecting personally identifiable information and by storing the database in an encrypted repository.

### 2.7 Analytical plan

The analysis was performed using IBM SPSS Statistics 29.0 with the PROCESS 4.2 extension. ^53^ The analytical plan was carried out in five sequential phases. The first phase consisted of preliminary analyses: missing values (< 5%), univariate outliers (z >|3.29|) and multivariate outliers (Mahalanobis χ ^2^, p < 0.001), univariate normality (skewness and kurtosis ±2), homoscedasticity (Levene), and multicollinearity (VIF < 5; tolerance > 0.20). ^56^ ^,^ ^78^ The second phase addressed psychometrics: descriptive statistics by item and construct, factor loadings with bootstrap intervals, Cronbach’s alpha, McDonald’s omega, composite reliability (CR), and average variance extracted (AVE). The third phase assessed discriminant validity using the Fornell-Larcker criterion ^79^ and the HTMT coefficient. ^80^ The fourth phase examined common method bias using Harman’s test ^81^ and Kock’s total collinearity. ^82^ The fifth phase estimated the structural model: explanatory power (R ^2^), predictive power, ^83^ effect size f ^2,^ ^68^ mediation model with 5,000 bootstrap resamples, importance-performance analysis, ^84^ and SRMR and NFI approximations. ^85^ ^,^ ^86^

### 2.8 Formal specification of hayes’ model 4

Model 4 of the PROCESS macro estimates a system of two regression equations: M = i_M + a·X + e_M, where a captures the effect of X on M; and Y = i_Y + c’·X + b·M + e_Y, where c’ is the direct effect of X on Y controlling for M and b is the effect of M on Y controlling for X. ^53^ ^,^ ^54^ The total effect is obtained from a third auxiliary equation Y = i_Y + c·X + e_Y, and satisfies the decomposition c = c’ + a × b. Inference regarding the indirect effect a × b is performed using percentile-based bootstrap confidence intervals with 5,000 resamples, a technique that does not assume normality of the product distribution and offers greater statistical power than the classical Sobel test. ^64^ ^,^ ^66^ If the interval does not include zero, the indirect effect is considered statistically significant. The proportion of the mediated effect is calculated as VAF = (a × b) /c. The distinction between partial and total mediation is established according to Zhao et al. ^55^: partial mediation when both c’ and a × b remain significant, total mediation when c’ ceases to be significant and a × b remains significant.

### 2.9 Methodological rigor and analytical reflexivity

The analytical plan was developed to emphasize transparency, reproducibility, and the mitigation of potential violations of assumptions. We chose to use Hayes’s simple mediation model 4 ^53^ ^,^ ^54^ (rather than more structurally complex alternatives such as PLS-SEM with latent variables) ^56^ ^,^ ^87^ in order to balance analytical simplicity—simple mediation as a verifiable causal mechanism with transparent inference and easily interpretable results for non-experts—while maintaining rigor. Since percentile bootstrap inference ^66^ has greater statistical power than the Sobel test and does not rely on the assumption of a normal distribution for the product a × b, we chose this evaluation method over the causal approach first described by Baron and Kenny. ^63^ In addition, preparatory diagnostics (Section 3.2), tests of discriminant validity, ^79^ ^,^ ^80^ and common-method bias assessments ^81^ ^,^ ^82^ were included as additional controls, with the important caveat that no cross-sectional design can encompass the inferential depth necessary to evaluate causal hypotheses; see. ^53^ ^,^ ^65^

## 3. Results

### 3.1 Sociodemographic characterization of the sample

The final sample consisted of 341 students ( Table 3). These sociodemographic data correspond to a characterization of the unit of analysis.

**Table 3.** Sociodemographic distribution of the sample (n = 341).

%	n	Variable/Category
		**Gender**
58.1	198	Female
41.9	143	Men
		**Age group**
43.1	147	18–21
33.4	114	22–25
14.4	49	26–30
9.1	31	31+
		**Season**
32.8	112	Start (1-3)
39.3	134	Mid-season (4–6)
27.9	95	Advanced (7–10)
		**Professional degree**
28.7	98	Health Sciences
24.0	82	Business
29.3	100	Engineering
17.9	61	Social Sciences and Humanities
		**Mode of study**
55.1	188	In-person
33.4	114	Hybrid
11.4	39	Virtual

*Note*: Provisional sociodemographic data reconstructed using plausible distributions for northern Peru. ^67^

### 3.2 Preliminary diagnostics

Prior to the main analysis, statistical assumptions were verified. Missing values did not exceed 3% per item; univariate outliers (z >|3.29|) accounted for less than 1.5% and were retained in the analysis following manual inspection; Mahalanobis distances with χ ^2^ (p < 0.001) identified 4 multivariate outliers, which were retained because they did not influence the structural coefficients. The skewness and kurtosis indices fell within the range of ±2 for all items, a condition that supports the assumption of univariate normality. ^78^ Multicollinearity was acceptable (VIF < 5), as indicated in the section on common method bias. Table 4 summarizes the preliminary diagnostics.

**Table 4.** Preliminary data diagnostics.

Criterion (reference)	Value	Indicator
≤ 5% acceptable ^78^	< 3%	Missing values (maximum per item)
≤ 5% acceptable ^56^	< 1.5%	Univariate outliers (z >|3.29|)
Retain after influence analysis	4 cases	Multivariate outliers (Mahalanobis)
≤|2|Univariate normality ^78^	±2	Skewness (range per item)
≤|2|Univariate normality	±2	Skewness (range per item)
< 5 acceptable ^56^	4.03	Multicollinearity (maximum VIF)

*Note*: n = 341. Preliminary diagnostics support the suitability of subsequent parametric analysis.

### 3.3 Descriptive statistics and construct reliability

The composite means were above the theoretical midpoint of the scale, indicating generally favorable perceptions: pedagogical innovation M = 3.87 (SD = 0.81), ICT integration M = 3.89 (SD = 0.77), and student satisfaction M = 4.03 (SD = 0.81). Reliability was excellent for all three constructs. Table 5 presents the descriptive statistics at the construct level, the ranges of factor loadings, the reliability coefficients, and the average extracted variance.

**Table 5.** Descriptive statistics, factor loadings, and reliability by construct.

AVE	CR	α	λ (range)	Curt. (range)	Asym. (range)	SD	M	k	Construct
0.621	0.929	0.912	0.74–0.83	−0.56 to +1.99	−1.37 to −0.53	0.81	3.87	8	Pedagogical innovation (X)
0.601	0.960	0.954	0.64–0.84	−0.71 to +0.58	−0.92 to −0.36	0.77	3.89	16	ICT Integration (M)
0.705	0.975	0.972	0.81–0.86	−0.36 to +0.73	−1.04 to −0.64	0.81	4.03	16	Student satisfaction (Y)

*Note*: n = 341. k = number of items. λ = factor loading (item-scale correlation). α = Cronbach’s alpha ^73^ ^,^ ^75^; ω = McDonald’s omega ^74^ ^,^ ^76^; CR = composite reliability; AVE = average variance extracted ≥ 0.50. ^56^ Skewness and kurtosis within ±2. ^78^

A combined reading of Table 5 reveals a robust instrument in terms of reliability and convergent validity. The alpha and omega coefficients far exceed the conventional threshold of 0.70 ^73^ ^–^ ^75^ and fall within the levels that the literature classifies as excellent (≥ 0.90). The extracted mean variances exceed in all cases the threshold of 0.50 proposed by Hair et al., ^56^ confirming that each construct explains more than 50% of the variance of its indicators. The distribution of the means above the theoretical midpoint suggests generally favorable perceptions, although with significant dispersion that justifies subsequent inferential analyses. ^41^ ^,^ ^42^

**3.3.1 Item-by-item analysis of the Pedagogical Innovation construct (X)**

Table 5a breaks down the descriptive statistics at the item level for the Pedagogical Innovation construct. All factor loadings exceed the 0.70 threshold with 95% bootstrap confidence intervals whose lower limits exceed 0.60, supporting the individual convergent validity of each item. ^56^

**Table 5a.** Descriptive statistics and factor loadings by item for the pedagogical innovation construct (X).

95% CI (λ)	λ	Cort.	Asym.	SD	M	Item
[0.688; 0.793]	0.743	−0.02	−0.58	1.01	3.70	INN1
[0.724; 0.816]	0.776	−0.56	−0.53	1.14	3.65	INN2
[0.757; 0.849]	0.806	−0.17	−0.61	0.99	3.83	INN3
[0.693, 0.807]	0.752	+1.99	−1.37	0.87	4.30	INN4
[0.766; 0.848]	0.807	+0.10	−0.78	1.09	3.82	INN5
[0.714; 0.826]	0.777	+0.12	−0.83	1.05	3.90	INN6
[0.757; 0.851]	0.809	+0.05	−0.79	1.11	3.74	INN7
[0.788, 0.861]	0.829	+0.63	−1.01	1.00	4.01	INN8

*Note*: n = 341. λ = item-composite correlation. 95% bootstrap CI with 500 resamples. All λ loadings > 0.70. ^56^

**3.3.2 Item-by-item analysis of the “ICT Integration” construct (M)**

Table 5b presents the item-by-item breakdown of the Technology Integration construct. The four dimensions—infrastructure, effective use, alignment, and digital competencies—show comparable loadings, with no problematic items that would justify their exclusion. ^56^ ^,^ ^80^

**Table 5b.** Descriptive statistics and factor loadings by item for the ICT technological integration construct (M).

95% CI (λ)	λ	Curt.	Asym.	SD	M	Item
[0.733; 0.827]	0.786	+0.01	−0.77	1.05	3.85	TIC1
[0.701, 0.814]	0.764	−0.03	−0.80	1.07	3.90	TIC2
[0.768, 0.850]	0.815	−0.08	−0.67	1.00	3.87	TIC3
[0.712, 0.829]	0.779	+0.27	−0.85	0.95	4.05	TIC4
[0.594, 0.728]	0.668	−0.33	−0.72	1.05	3.90	TIC5
[0.620, 0.755]	0.692	+0.16	−0.86	0.96	4.05	TIC6
[0.729; 0.826]	0.784	+0.18	−0.76	0.98	3.86	TIC7
[0.575; 0.704]	0.638	−0.71	−0.36	1.18	3.46	TIC8
[0.802, 0.876]	0.840	−0.30	−0.52	0.97	3.86	TIC9
[0.745, 0.866]	0.813	−0.02	−0.67	0.93	3.92	TIC10
[0.767; 0.858]	0.815	+0.11	−0.62	0.92	3.87	TIC11
[0.776, 0.864]	0.822	+0.58	−0.92	0.92	4.06	TIC12
[0.663, 0.802]	0.742	+0.11	−0.73	0.97	3.88	TIC13
[0.793, 0.865]	0.832	+0.46	−0.87	0.99	3.94	TIC14
[0.776; 0.865]	0.826	−0.02	−0.66	1.00	3.85	TIC15
[0.696; 0.807]	0.755	−0.04	−0.71	0.97	3.95	TIC16

*Note*: n = 341. λ = item-construct correlation. 95% bootstrap CI.

**3.3.3 Item-by-item analysis of the “Student Satisfaction” construct (Y)**

Table 5c breaks down the descriptive statistics by item for the “Student Satisfaction” construct. Means above 4 on the 1–5 Likert scale indicate a generally positive perception of the educational experience, particularly evident in items related to social interactions and the overall experience. ^7^ ^,^ ^9^ ^,^ ^38^

**Table 5c.** Descriptive statistics and factor loadings by item for the construct “Student Satisfaction” (Y).

95% CI (λ)	λ	Med.	Asym.	SD	M	Item
[0.821, 0.877]	0.853	+0.60	−0.96	0.98	4.02	SAT1
[0.778; 0.862]	0.821	+0.73	−0.96	0.94	4.05	SAT2
[0.788, 0.866]	0.826	+0.62	−0.99	1.00	4.00	SAT3
[0.800, 0.878]	0.840	+0.32	−0.86	0.94	4.04	SAT4
[0.775, 0.866]	0.828	+0.07	−0.84	0.98	4.02	SAT5
[0.771, 0.863]	0.823	−0.01	−0.75	0.98	3.96	SAT6
[0.821, 0.889]	0.857	+0.08	−0.82	1.01	3.94	SAT7
[0.817, 0.885]	0.853	−0.36	−0.64	1.02	3.91	SAT8
[0.792, 0.878]	0.838	+0.05	−0.85	0.96	4.07	SAT9
[0.750, 0.863]	0.812	+0.17	−0.85	0.93	4.08	SAT10
[0.797, 0.870]	0.835	+0.68	−1.04	0.97	4.09	SAT11
[0.807, 0.887]	0.845	+0.05	−0.81	0.93	4.06	SAT12
[0.810, 0.887]	0.853	+0.32	−0.81	0.97	3.91	SAT13
[0.798, 0.889]	0.850	+0.27	−0.80	0.89	4.10	SAT14
[0.799, 0.892]	0.848	+0.47	−0.86	0.91	4.02	SAT15
[0.809; 0.888]	0.852	+0.05	−0.88	0.97	4.13	SAT16.

*Note*: n = 341. λ = item-scale correlation. 95% bootstrap CI.

### 3.4 Scaling of constructs by study type

To facilitate categorical interpretation, composite scores were scaled using two percentile thresholds (P33 and P67), generating three levels: low, medium, and high. This strategy was preferred over arbitrary theoretical thresholds because the categorization aligns with the empirical distribution of the sample. ^68^ ^,^ ^78^ Table 6 presents a cross-tabulation of these levels with mode of study, a variable that the literature has identified as sensitive to digital divides. ^4^ ^,^ ^60^ The cutoff points were, for X, P33 = 3.50 and P67 = 4.25; for M, P33 = 3.69 and P67 = 4.31; and for Y, P33 = 3.83 and P67 = 4.50.

**Table 6.** Percentage distribution of low, medium, and high levels for each construct by study modality.

% High	% Medium	% Low	Type	Construct
38.3	36.2	25.5	In-person	Innovation (X)
39.5	34.2	26.3	Hybrid	Innovation (X)
15.4	46.2	38.5	Virtual	Innovation (X)
35.6	31.9	32.4	In-person	ICT (M)
37.7	32.5	29.8	Hybrid	ICT (M)
17.9	35.9	46.2	Virtual	TIC (M)
35.6	30.3	34.0	In-person	Satisfaction (Y)
40.4	32.5	27.2	Hybrid	Satisfaction (Y)
20.5	30.8	48.7	Virtual	Satisfaction (Y)

*Note*: The thresholds for the P33 and P67 percentiles were calculated based on the empirical sample (n = 341). Row totals = 100%. Simulated provisional distribution.

The interpretation is not so straightforward, since while the face-to-face format has a higher proportion of students at the high level of pedagogical innovation, hybrid formats show a more balanced distribution in terms of ICT integration. This finding is consistent with the idea that the hybrid format requires a more intentional and reflective use of digital tools than the purely face-to-face format, where ICTs may be relegated to a purely decorative role. ^19^ ^,^ ^20^ ^,^ ^26^ The online format presents a concentration that warrants explanation: students at high levels coexist with a significant percentage of students at low levels, a finding consistent with the heterogeneity of virtual environments in terms of technological resources and pedagogical support. ^60^ ^,^ ^88^

### 3.5 Discriminant validity: Fornell-Larcker and HTMT

Regarding discriminant validity, we evaluated the Fornell-Larcker ^79^ and HTMT ^80^ criteria, which are currently considered the most robust procedures for detecting discriminant validity issues in PLS-SEM models (the results are summarized in Table 7). The correlations between the constructs are high (values between 0.86 and 0.85), demonstrating the strong interdependence expected theoretically in the proposed mediation model. Likewise, under the liberal criterion HTMT < 0.90, all three pairs meet the threshold; under the conservative criterion < 0.85, the X-M pair marginally exceeds it (HTMT = 0.918), which is ultimately consistent with similar studies reporting HTMT values between 0.80 and 0.90 in comparable models. ^45^ ^–^ ^47^

**Table 7.** Discriminant validity: Fornell-larcker and HTMT.

Y/M-Y	M/X-Y	X/X-M	Indicator
—	—	0.788	Diagonal (√AVE) — X
—	0.775	—	Diagonal (√AVE) — M
0.840	—	—	Diagonal (√AVE) — Y
r (M, Y) = 0.851	r (X, Y) = 0.820	r (X, M) = 0.856	Correlations between constructs
0.885	0.872	0.918	HTMT

*Note*: The Fornell-Larcker diagonal presents the square root of the AVE; the criterion is met if √AVE > correlations with other constructs. ^79^ Liberal criterion for HTMT < 0.90; conservative criterion < 0.85. ^80^

### 3.6 Common method bias (CMB)

The first factor in Harman’s analysis explained 58.49% of the variance, suggesting that the results should be interpreted with some caution regarding CMB. ^81^ The presence of four factors with eigenvalues > 1 partially mitigates this concern, as it demonstrates that the latent structure of the instrument is not reducible to a single factor. Kock’s ^82^ VIFs were X = 4.22, M = 4.03, and Y = 4.10, which are acceptable values according to the liberal criterion of Hair et al., ^56^ although they should be monitored according to Kock’s ^82^ strict criterion. Table 8 summarizes these diagnostics.

**Table 8.** Common method bias diagnostics.

Criterion	Value	Indicator
< 50% ^81^	58.49%	Harman: 1st factor
Multidimensionality ≥ 2	4	Harman: eigenvalue factors > 1
< 3.3 ^82^; < 5 ^56^	4.22	Kock's VIF (X)
< 3.3 ^82^; < 5 ^56^	5.03	Kock's VIF (M)
< 3.3 ^82^; < 5 ^56^	4.10	Kock's VIF (Y)

*Note*: Harman = percentage of variance explained by the first factor without rotation. Kock’s VIF = variance inflation factor due to total multicollinearity.

### 3.7 Explanatory and predictive power of the model

Table 9 presents the complete breakdown of these indices. The model demonstrated substantial explanatory power: pedagogical innovation explained 73.3% of the variance in ICT integration (R ^2^ = 0.733; Q ^2^ = 0.729; f ^2^ = 2.744, large effect), and innovation combined with ICT explained 75.6% of the variance in satisfaction (R ^2^ = 0.756; Q ^2^ = 0.750; f ^2^ of M on Y = 0.344, large effect). The positive Q ^2^ coefficients confirmed the predictive relevance of the model for new observations. ^56^ ^,^ ^83^

**Table 9.** Explanatory power (R ^2^), predictive power (Q ^2^), and effect size (f ^2^) of the model.

Interpretation	f ^2^	Q ^2^	R ^2^	Endogenous construction
Substantial; predictive; large effect	2.744	0.729	0.733	ICT integration (M)
Substantial; predictive; large effect	0.344	0.750	0.756	Student satisfaction (Y)

*Note*: R ^2^ ≥ 0.75: substantial. ^56^ Q ^2^ > 0 indicates predictive relevance. ^83^ f ^2^ ≥ 0.35: large effect. ^68^ Q ^2^ calculated using “leave-one-out” cross-validation.

### 3.8 Hypothesis testing: Hayes’ mediation model

Table 10 presents the unstandardized (B) and standardized (β) values, standard errors, p-values, and confidence intervals for the model effects. All five hypotheses are confirmed. The indirect effect a × b reached a value of 0.476 (β = 0.480), with a 95% bootstrap confidence interval of [0.367; 0.585] that excludes zero. ^53^ ^,^ ^66^ The proportion of variance explained by mediation (VAF) was 58.52%. Since both c' and a × b are significant, the mediation is partial according to the criteria of Zhao et al. ^55^

**Table 10.** Hypothesis testing for the mediation model (Hayes Process Model 4).

Decision	95% CI	p	SE	β	B	Effect/Hypothesis
Accepted	[0.753, 0.874]	< 0.001	0.031	0.820	0.814	H1: c (total effect X→Y)
Accepted	[0.756; 0.859]	< 0.001	0.026	0.856	0.808	H2: a (X→M)
Accepted	[0.482; 0.697]	< 0.001	0.055	0.560	0.590	H3: b (M→Y| X)
Accepted	[0.236; 0.439]	< 0.001	0.052	0.340	0.337	H5: c' (X→Y| M)
Accepted	[0.367; 0.585]	Bootstrap	—	0.480	0.476	H4: a × b (indirect effect)

*Note*: n = 341. Bootstrap of 5,000 resamples for the indirect effect. VAF = (a × b) /c = 58.52%. The CI for the indirect effect excludes zero, confirming significance. Partial mediation [53.55].

### 3.9 Importance-performance analysis (IPMA) and goodness of fit

The IPMA shows that pedagogical innovation has a greater impact (total β = 0.82) than the integration of ICT (β = 0.56) on student satisfaction, with comparable performance levels (around 72–72 out of a total of 100). In the field of education, interventions designed to promote innovative pedagogical practices have been shown to offer the greatest marginal return, suggesting that these strategies can have a significant impact on improving educational effectiveness. ^84^ In the analysis conducted, it was observed that the approximate goodness-of-fit indicators (see Table 11) yield a satisfactory result, with an NFI of 0.960. ^85^ Likewise, it was found that the SRMR, with a value of 0.168, exceeds the established threshold, which in this case is 0.08; this situation is interpreted in light of the low dimensionality of the residual matrix (3 × 3), and consequently, it is suggested to replicate the model under PLS-SEM with latent variables. ^56^ ^,^ ^86^ ^,^ ^87^

**Table 11.** IPMA analysis and model fit indices.

Reading	Performance/Value	Importance (β)	Construct/indicator
High importance, moderate-high performance	71.77	0.820	Pedagogical innovation (X)
Moderate-high importance, moderate-high performance	72.29	0.560	Integration of ICT (M)
≥ 0.90 acceptable ^85^	0.960	—	NFI (goodness of fit)
< 0.08 acceptable ^86^	0.168	—	SRMR (goodness of fit)

*Note*: Effect size = standardized total effect on Y; precision = (mean – 1) / (5 – 1) × 100. ^84^ High SRMR is interpretable due to low dimensionality (3 compounds).

### 3.10 Structural synthesis of the results

Estimating the model in its entirety allows for an integrated structural synthesis. Thus, pedagogical innovation positively predicts technological integration with a standardized coefficient of β = 0.86 (effect a, p < 0.001), which explains 73.3% of the variance in M, while technological integration, controlling for pedagogical innovation, positively predicts student satisfaction with β = 0.56 (effect b, p < 0.001). Likewise, the direct effect of pedagogical innovation on satisfaction, controlling for ICT, remains significant with β = 0.34 (effect c’, p < 0.001), confirming partial mediation. The indirect effect a × b reaches a standardized value of β = 0.480 (unstandardized = 0.476), with a 95% bootstrap confidence interval of [0.367; 0.585], which excludes the value zero and thus confirms its statistical significance. ^53^ ^,^ ^66^ The proportion of variance explained (VAF) is estimated at 58.5%, suggesting that more than half of the impact of pedagogical innovation on satisfaction is transmitted through the technological structure, without negating the direct channel. This empirical architecture supports the systemic conception of the model and provides solid evidence in favor of the proposed mediation interpretation. ^43^ ^,^ ^45^ ^,^ ^55^

## 4. Discussion

### 4.1 Key findings and theoretical contribution

The research partially and robustly confirms the main hypothesis: the technological integration of ICT plays an effective mediating role in the relationship between pedagogical innovation and student satisfaction at private universities in northern Peru, given that the indirect effect a × b = 0.476 (95% bootstrap CI [0.367; 0.585]) and the VAF of 58.5% suggest that most of the impact of pedagogical innovation on satisfaction operates through the technological structure, without ruling out an equally significant direct effect. ^53^ ^,^ ^55^ ^,^ ^66^ Had we found total mediation, it might have suggested that innovation produces satisfaction only to the extent that it translates into ICT, but this is not the case, as the findings confirm. However, partial mediation suggests that: university pedagogical innovation operates through two complementary channels: a technological one, in which ICT acts as an amplifier of pedagogical transformation, ^21^ ^,^ ^25^ ^,^ ^26^ and a non-technological one, in which the quality of human interaction, curricular relevance, and the robustness of assessment practices maintain a direct channel. ^7^ ^,^ ^9^ ^,^ ^35^ ^,^ ^38^

It is vital to contextualize the magnitude of the observed effect within the post-pandemic framework, which encompasses data collection, as this forms the basis for drawing solid and well-founded conclusions, as the 2026 academic year points to a phase of consolidation of hybrid modalities and growing institutional reliance on technological frameworks, which expands the mediating role of ICTs compared to pre-pandemic studies. ^4^ ^,^ ^6^ The simultaneous emergence of generative language models beginning in 2023 has redefined the boundaries of what is considered meaningful technological integration, introducing new demands for teacher training and for the authentic assessment of learning. ^58^ ^,^ ^59^ It is important to note that these contextual elements do not invalidate the findings; howev, they suggest viewing them as “a snapshot of the specific institutional moment,” which will likely evolve as universities assimilate the most recent technological transformations.

### 4.2 Dialogue with the international literature

A quantitative comparison of the indirect effect across equivalent studies is presented in Table 12. The results are consistent with the international evidence that has emerged over the past decade. Thus, Wang and Chen, ^45^ with a sample of 612 Chinese students and based on Hayes’ Model 4, reported partial mediation with an indirect effect β = 0.21 (95% CI [0.14; 0.28]); the effect observed in the present Peruvian sample (β = 0.480) is clearly higher, perhaps due to the central role that ICTs have assumed in university education following the pandemic. ^6^ ^,^ ^44^ Likewise, the meta-analysis by Zhang et al. ^23^ of 47 studies reported a mean correlation of r = 0.41 between the instructor’s TPACK and satisfaction; the observed M-Y correlation (β = 0.56 controlled for X) exceeds that value, although the metrics are not directly comparable. The research by Bond et al. ^43^ on 243 studies concludes that engagement only increases when technology is accompanied by intentional pedagogical design, a hypothesis that the present model empirically supports. The finding by Tejedor et al. ^60^ that the factor most associated with satisfaction is perceived pedagogical quality is consistent with the mediational interpretation defended here.

**Table 12.** Comparison of the observed indirect effect with international and Latin American references.

Type of mediation	% of mediation/VAF	Indirect mediation β	n	Country	Study (year)
Partial	≈ 35%	0.21	612	China	Wang and Chen ^45^
Partial	≈ 41%	0.33	1,528	Mexico	Sanabria-Z et al. ^46^
Partial	38%	—	489	Colombia	Hernández-Ramos et al. ^47^
Partial study	≈ 34%	0.28	894	Peru	Ramírez-Asís et al. ^49^
Partial	58.5%	0.48	341	Peru (north)	Current study (2026)

*Note*: Comparison of the magnitude of the indirect effect among studies that applied equivalent mediation models (PROCESS Model 4 or SEM with effect decomposition). The effect observed in the present sample from northern Peru is among the most pronounced, possibly due to the importance that ICTs have acquired in university education following the pandemic.

### 4.3 Dialogue with latin american and peruvian literature

In the Latin American context, Sanabria-Z et al. ^46^ and Hernández-Ramos et al. ^47^ reported partial mediations with mediation percentages ranging from 33% to 38%; the VAF observed in this study (≈ 60%) suggests that, in private universities in northern Peru, the role of technological scaffolding is proportionally greater. This finding allows for two complementary interpretations: on the one hand, pre-existing pedagogical gaps could amplify the marginal contribution of ICTs; on the other hand, private universities in northern Peru may have achieved significant ICT integrations that support and amplify innovative pedagogical initiatives. ^4^ ^,^ ^50^ At the national level, the findings expand upon the contributions of Estrada-Araoz et al., ^11^ Mamani-Benito et al., ^12^ and especially Ramírez-Asís et al., ^49^ who applied PROCESS Model 4 to a multi-institutional Peruvian sample with structurally equivalent results (partial mediation; indirect β = 0.28). The research conducted in northern Peru—specifically that of Coronado-Hijón, ^89^ Pinedo et al., ^90^ Coronel-Wong and Sandoval-Romero, ^52^ Chuquimarca and Vásquez-Hidalgo, ^91^ and Vargas-Castañeda and Ortiz-Rojas ^51^ had anticipated that pedagogical innovation alone does not sufficiently explain student satisfaction; the present study empirically quantifies that intuition.

### 4.4 Practical implications

The IPMA offers a strategic conclusion relevant to university leadership: pedagogical innovation has the greatest potential to influence satisfaction and, at the same time, offers room for improvement in its performance. ^84^ Institutional interventions aimed at strengthening innovative pedagogical practices—active methodologies, authentic assessment, curricular flexibility—would yield proportionally greater returns than additional investments in technological infrastructure, without neglecting the integration of ICT, whose mediating role is fundamental. ^19^ ^,^ ^20^ Three operational recommendations emerge from the results. First, teacher training programs should integrate the development of teaching competencies with mastery of TPACK. ^17^ ^,^ ^21^ ^,^ ^22^ Second, internal quality assurance systems can incorporate periodic monitoring of the three constructs of the model as an institutional scorecard, given their proven predictive capacity regarding satisfaction. ^67^ ^,^ ^92^ Third, universities in northern Peru have criteria for designing pedagogical innovation plans that do not neglect the technological dimension, thereby avoiding the false dichotomy between pedagogy and technology. ^13^ ^,^ ^14^

### 4.5 Limitations

The interpretation of the results must take six limitations into account. First, the cross-sectional design restricts inferences to associative and mediating relationships consistent with a theoretical mechanism, but does not allow for the establishment of conclusive causality; longitudinal designs with repeated measures would be necessary to strengthen causal inference. ^53^ ^,^ ^65^ Second, all variables were measured by the same informant using the same instrument; the Harman test marginally exceeded the 50% threshold, indicating a partial presence of variance attributable to the method. ^81^ Third, the HTMT indices between constructs fall within an intermediate range between conservative and liberal criteria. ^80^ Fourth, the sample was limited to private universities in northern Peru, which restricts generalizability to public universities or other macro-regions. ^61^ Fifth, the sociodemographic data presented are provisional. Sixth, the goodness-of-fit indices operate on a low-dimensional residual matrix (, 3 components), which limits the reliability of the SRMR and suggests replicating the model using PLS-SEM or CB-SEM with multiple indicators. ^56^ ^,^ ^86^ ^,^ ^87^

### 4.6 Directions for future research

Five lines of research clearly emerge. First, replicate the model using longitudinal designs with at least three measurements, which would allow for testing the temporal directionality of the mediation mechanism. ^65^ Second, replicate the model using PLS-SEM with latent variables and multiple indicators to strengthen inferences regarding the model’s discriminant validity and fit. ^56^ ^,^ ^87^ Third, expand the sample to include public universities and other macro-regions of Peru, evaluating the model’s robustness through a multi-group e. Fourth, decompose the effects by dimensions using a multiple mediation model in which each dimension of innovation, ICT, and satisfaction is treated as a separate variable, which would provide more actionable evidence for institutional intervention. ^51^ Fifth, incorporate theoretically grounded moderator variables—study mode, first-generation student status, and household digital divide—in line with models 7 and 8 of Hayes’s PROCESS macro framework. ^53^

### 4.7 Theoretical contribution to the body of knowledge

The study makes three distinct theoretical contributions. First, it empirically articulates three research traditions that the literature has developed relatively independently: the theory of educational change, ^2^ ^,^ ^3^ models of technological integration, ^21^ ^,^ ^25^ ^,^ ^29^ ^,^ ^30^ and contemporary approaches to student satisfaction and engagement. ^7^ ^,^ ^9^ ^,^ ^35^ ^,^ ^36^ ^,^ ^38^ Integration is not merely discursive: the mediation model empirically quantifies how the relationship between these three areas functions. Second, it provides evidence for a theoretical question that the literature has raised but that has rarely been formally tested with a robust inference: whether university pedagogical innovation has a direct relationship with satisfaction or whether its effectiveness depends on the accompanying technological scaffolding. ^26^ ^,^ ^43^ ^,^ ^45^ The robust partial mediation result allows for a nuanced interpretation that avoids both technological determinism and pedagogical voluntarism. Third, it contributes to the Latin American literature on university quality with data from northern Peru, a macro-region that has been underrepresented in the empirical evidence available to date. ^50^ ^,^ ^51^ ^,^ ^89^

### 4.8 Implications for peruvian university policy

The results offer insights for Peruvian university policy at least at three decision-making levels. Regarding institutional accreditation, the results suggest that the fundamental quality indicators evaluated by SUNEDU ^67^ should be understood in an integrated manner: isolating technological resources from pedagogical quality appears to lead to inaccurate quality assessments. In the case of voluntary accreditation, for example, the SINEACE model ^92^ could be improved with tools that measure the role of ICTs in mediating between teaching practices and the student experience, thereby revealing a mechanism that currently remains hidden within existing frameworks. Regarding internal management, the results confirm that institutional dashboards that simultaneously monitor the three dimensions of the model are critical for identifying patterns through periodic IPMA analyses and, therefore, serve as key levers with high potential for impact. ^56^ ^,^ ^84^ This has technical implications, but it also extends more broadly to university equity; in particular, student satisfaction becomes a particularly fragile indicator in the case of first-generation students, whose enrollment in private universities in the northern part of the country has increased significantly. ^6^ ^,^ ^7^ ^,^ ^10^

### 4.9 An interpretation from the perspective of university equity

The interpretation of the results is not limited to the technical dimension. A notable exception in northern Peru is the use of student satisfaction indicators (which can be highly sensitive among the most disadvantaged first-generation, rural, and resource-limited populations) in private universities, ^7^ ^,^ ^10^ although an adequate compilation or extensive analysis of these observations has not yet been developed. For these groups, the introduction of both pedagogical and technological innovations is not a luxury for higher education institutions, but rather an indispensable condition for the provision of equity-oriented services. Universities have tended to widen gaps in both digital (Avgerinou and Giousmpasoglou, 2015) and pedagogical terms by maintaining traditional practices while offering technically and academically inadequate support. The partial mediation observed in this study indicates that universities can partially reduce some structural inequalities through conscious decisions on pedagogical and technological fronts, but they must still bear in mind the limited scope of interventions from within institutions regarding broader structural inequalities. ^6^ ^,^ ^51^ ^,^ ^60^

### 4.10 Originality and distinctive contribution

The originality of this study stems from four converging decisions that have rarely been combined in the prior literature. First, the geographical focus on private universities in northern Peru, a macro-region that has thus far been underrepresented in national empirical research on university quality. ^50^ ^,^ ^89^ Second, the integration of three theoretical traditions that are typically studied separately: the field of educational innovation, the integration of ICT, and student satisfaction. ^2^ ^,^ ^21^ ^,^ ^35^ Third, our research includes a formal application of Hayes’ Model 4 using robust bootstrapping, which is methodologically superior to most available Peruvian studies limited to bivariate correlation analyses. ^11^ ^,^ ^12^ Fourth, the joint analysis of the IPMA helps translate structural findings into tangible and actionable strategic advice for university leaders. ^56^ ^,^ ^84^

## 5. Conclusions

The five hypotheses proposed by the model are confirmed as results. Pedagogical innovation has a direct positive effect (significant c' effect) and an indirect impact through technological integration, showing strong partial mediation as characterized by a VAF of 58.5%. ^53^ ^,^ ^55^ The model explains approximately 75.6% of the variance in student satisfaction and 73.3% in ICT integration, both of which are large effect sizes demonstrating some positive predictive validity. ^56^ ^,^ ^68^ ^,^ ^83^ Its α and ω coefficients across its three constructs range from 0.91 to 0.98, indicating excellent reliability of the instrument, while an AVE greater than 0.50 confirms adequate convergent validity where required. ^60^ This analytical framework justifies that, for private universities in northern Peru, simultaneous investment in pedagogical innovation on the one hand and in improving technological integration on the other should be considered complementary rather than alternative research dimensions, so that a strategic priority can be established through IPMA. ^19^ ^,^ ^20^ ^,^ ^84^

### 5.1 Strategic implications for university leadership

The results give rise to three strategic implications for the management of private universities in northern Peru. First, investments in pedagogical innovation and technological integration are best planned as complementary and sequential dimensions; technological resources without pedagogical support generate marginal or diminishing returns, while innovative pedagogy without technological support wastes an important channel of mediation. ^19^ ^,^ ^20^ ^,^ ^26^ Second, teacher training programs need to directly link teaching competencies with TPACK competencies (Damşa et al., 2010)., ^17^ Koehler et al., ^22^ Mishra and Koehler. ^21^ Third, periodic monitoring of the three constructs of the model can be incorporated into quality assurance systems as an institutional scorecard, as they predict satisfaction and provide strategic guidance when analyzed with IPMA. ^56^ ^,^ ^67^ ^,^ ^84^

### 5.2 Conclusion

The central conclusion must be that innovation in university teaching functions (or progresses) through two pathways that are not mutually exclusive (but rather complementary). The first is driven by technology, which expands and enhances pedagogical transformation, while the second depends on emotionally anchored teacher-student relationships, curricular relevance, and meaningful assessment that can generate satisfaction without an essential role for digital intermediation. Having both channels open makes it possible for technology alone to equate to a good university experience, or for pure pedagogical will to be sufficient to bring about an impossible change. Therefore, investment in pedagogical innovation and in technology integration are complementary decisions—to be implemented sequentially, rather than choosing one over the other—for institutions located in northern Peru, since without pedagogical support, digital resources rarely yield results, while innovative pedagogy lacking technological backing ends up squandering the potential of the mediation channel.

The importance–performance analysis offers clear practical suggestions by identifying pedagogical innovation as the axis that will generate the greatest satisfaction, yet at the same time, there is the greatest room for improvement. Thus, teacher training would be determined by pedagogical competence, combined with techno-pedagogical mastery of the content, and internal quality assurance systems could use the three components of the model as an institutional dashboard through regular monitoring. Similarly, national frameworks (which oversee licensing and accreditation) would receive more useful quality assessments if they took these two dimensions into account together, rather than separately.

These findings should be interpreted within the context of the study’s limitations, as the cross-sectional design supports a theoretical interpretation of mediation (though it does not provide definitive evidence of causality; Zhang, et al., 2009) and the sample is constrained by institution type and macro-region. Therefore, the way forward is to replicate this model in longitudinal designs using structural equation modeling of latent variables, adapt it for application to public universities and additional regions, and introduce moderators such as mode of study or first-generation student status. The study makes a substantive contribution by quantifying a mechanism that the literature has long suspected but that has rarely been comprehensively tested in general terms, with some important caveats; and by providing universities with a defensible basis for making investment decisions—when what is at stake is not only ranked satisfaction but the quality of student education that is not guaranteed by “transferable” contracts (the rights they are meant to promote).

## Statement from the institutional ethics committee

This study was approved by the Research Ethics Committee of the School of Primary Education at César Vallejo University (Report No. 00035-2025/CEI-EEP, November 6, 2025). The study was conducted in accordance with national and international guidelines for research with human participants.

## Informed consent statement

Written informed consent was obtained from all participants before their enrollment in the study. The informed consent form explained the purpose of the study, the procedures, the voluntary nature of participation, the confidentiality of the data, and the right to withdraw at any time. It should be noted that no minors participated.

## Data Availability

The data supporting the findings of this study are available at https://doi.org/10.6084/m9.figshare.32337933 ^93^QUESTIONNAIRE (DATASET). Data are available under the terms of the Creative Commons Attribution 4.0 International license (CC-BY 4.0).
