# Supplementary material for: The Mediating Role of ICTs in the Relationship Between Pedagogical Innovation and Student Satisfaction in Private Universities in Northern Peru
Source: F1000Res. 2026 Sep 21;15:1016. Originally published 2026 Jun 25. [Version 2] doi: 10.12688/f1000research.183195.2 (PMC13601789; doi:10.12688/f1000research.183195.2)
Supplement: Peer review history

# Reviewer report: Reviewer response for version 2

**DOI:** 10.5256/f1000research.209426.r523221

**Published:** 2026-09-23

**Authors:** Jose Carlos Montes Ninaquispe

**Affiliations:** 1 Universidad de San Martín de Porres, Chiclayo, Peru

I thank the authors for the revisions made to the manuscript. The revised version clarifies the contribution of the study, corrects the reporting of several numerical results, and more explicitly acknowledges the limitations concerning discriminant validity, common method bias, and the interpretation of model fit. These changes improve the transparency of the manuscript and provide a clearer context for interpreting its findings.

 Taking into account the revisions and the limitations now acknowledged, my assessment of this version is favorable. I approve the revised article and have no further comments or requests for revision.

Is the work clearly and accurately presented and does it cite the current literature?

Yes

If applicable, is the statistical analysis and its interpretation appropriate?

Yes

Are all the source data underlying the results available to ensure full reproducibility?

Yes

Is the study design appropriate and is the work technically sound?

Yes

Are the conclusions drawn adequately supported by the results?

Yes

Are sufficient details of methods and analysis provided to allow replication by others?

Yes

Reviewer Expertise:

Research professor and columnist in Scopus. Ph.D. in Research and Administration.

I confirm that I have read this submission and believe that I have an appropriate level of expertise to confirm that it is of an acceptable scientific standard.

# Reviewer report: Reviewer response for version 1

**DOI:** 10.5256/f1000research.202212.r501300

**Published:** 2026-08-20

**Authors:** Jose Carlos Montes Ninaquispe

**Affiliations:** 1 Universidad de San Martín de Porres, Chiclayo, Peru

**1. Sharpen the research gap.** The stated gap—that most Peruvian studies rely on bivariate analyses and rarely model the underlying mechanism—is partly undercut by the authors' own citation of Ramírez-Asís et al. (49), who already applied PROCESS Model 4 to a Peruvian sample with a structurally equivalent partial-mediation result. The authors should reposition the contribution more precisely (e.g., the first probabilistic, stratified test of this mechanism in the underrepresented northern macro-region, and the first to quantify the mediated proportion via VAF and translate it into IPMA-based management priorities) so that the novelty does not rest on a claim the manuscript itself contradicts.

**2. Substantiate the "problematic reality" with concrete data.** The Introduction establishes the problem rhetorically (structural inequality, "anachronistic" pedagogies, a "vulnerable" first-generation profile) but provides almost no empirical figures to demonstrate its magnitude. The argument would be considerably stronger with verifiable national and regional statistics—dropout/attrition rates in Peruvian private universities, the regional digital divide (e.g., household internet/device access in La Libertad, Lambayeque, Piura, and Áncash), and the actual share of first-generation students—rather than adjectival characterizations. At present only the population estimate (~145,000) is quantified.

**3. Strengthen and defend the statistical treatment.** Several diagnostics need a more transparent defense. The SRMR (0.168) is more than double the 0.08 threshold; the low-dimensional residual-matrix justification is reasonable but insufficient on its own, and running the recommended PLS-SEM replication now (rather than deferring it to future work) would resolve the concern directly. The HTMT for the innovation–ICT pair (0.918) exceeds the conservative criterion and, together with the very high inter-construct correlations (~0.86) and the a-path (β = 0.856), raises a discriminant-validity question the authors should address explicitly (e.g., reporting the HTMT confidence interval). Finally, Harman's single factor (58.49%) exceeds 50% and Kock's VIFs (up to 4.22) exceed the strict 3.3 criterion, so common-method bias should be acknowledged as a genuine (not merely cautionary) limitation, ideally supplemented by a marker-variable or unmeasured latent-factor test.

Is the work clearly and accurately presented and does it cite the current literature?

Yes

If applicable, is the statistical analysis and its interpretation appropriate?

Yes

Are all the source data underlying the results available to ensure full reproducibility?

Yes

Is the study design appropriate and is the work technically sound?

Yes

Are the conclusions drawn adequately supported by the results?

Yes

Are sufficient details of methods and analysis provided to allow replication by others?

Yes

Reviewer Expertise:

Research professor and columnist in Scopus. Ph.D. in Research and Administration.

I confirm that I have read this submission and believe that I have an appropriate level of expertise to confirm that it is of an acceptable scientific standard, however I have significant reservations, as outlined above.

# Reviewer report: Reviewer response for version 1

**DOI:** 10.5256/f1000research.202212.r501292

**Published:** 2026-08-20

**Authors:** Heyner Yuliano Marquez Yauri

**Affiliations:** 1 Universidad Nacional de Trujillo - UNT, Trujillo, Peru

The article addresses a topical and relevant issue in higher education: the relationship between pedagogical innovation, the integration of information and communication technologies, and student satisfaction at private universities in northern Peru. It highlights the development of a mediation model with a broad theoretical framework, the use of bootstrapping to estimate the indirect effect, and the aim of offering implications applicable to university management. The manuscript reports a sample of 341 students, a 40-item questionnaire and a partial mediation of ICT between pedagogical innovation and satisfaction.

 However, the manuscript requires a review of certain aspects relating to the traceability and nature of the data (note in Table 3); this issue must be clarified. Furthermore, the methodological approach adopted could be justified more convincingly. Finally, it is emphasised that the study provides valuable, contextualised evidence on the university experience and the management of educational innovation in northern Peru.

Is the work clearly and accurately presented and does it cite the current literature?

Yes

If applicable, is the statistical analysis and its interpretation appropriate?

Yes

Are all the source data underlying the results available to ensure full reproducibility?

Yes

Is the study design appropriate and is the work technically sound?

Yes

Are the conclusions drawn adequately supported by the results?

Yes

Are sufficient details of methods and analysis provided to allow replication by others?

Yes

Reviewer Expertise:

Education, sustainability, administration and business

I confirm that I have read this submission and believe that I have an appropriate level of expertise to confirm that it is of an acceptable scientific standard.
